# State Support: A Prerequisite for Global Health Network Effectiveness: Comment on "Four Challenges that Global Health Networks Face"

**DOI:** 10.15171/ijhpm.2017.86

**Published:** 2017-07-24

**Authors:** Robert Marten, Richard D. Smith

**Affiliations:** Faculty of Public Health and Policy, London School of Hygiene and Tropical Medicine, London, UK.

**Keywords:** Global Health, Health Policy, Global Health Networks

## Abstract

Shiffman recently summarized lessons for network effectiveness from an impressive collection of case-studies. However, in common with most global health governance analysis in recent years, Shiffman underplays the important role of states in these global networks. As the body which decides and signs international agreements, often provides the resourcing, and is responsible for implementing initiatives all contributing to the prioritization of certain issues over others, state recognition and support is a prerequisite to enabling and determining global health networks’ success. The role of states deserves greater attention, analysis and consideration. We reflect upon the underappreciated role of the state within the current discourse on global health. We present the tobacco case study to illustrate the decisive role of states in determining progress for global health networks, and highlight how states use a legitimacy loop to gain legitimacy from and provide legitimacy to global health networks. Moving forward in assessing global health networks’ effectiveness, further investigating state support as a determinant of success will be critical. Understanding how global health networks and states interact and evolve to shape and support their respective interests should be a focus for future research.

## Introduction

Shiffman recently summarized lessons for network effectiveness from an impressive collection of case-studies across tobacco use and alcohol harm, maternal and neonatal mortality, early childhood development and surgically-treatable conditions as well as tuberculosis and pneumonia.^1^ The networks involved in these areas, and their effectiveness, matter as they contribute to the shaping and framing of areas competing for attention and resources in global health. Recognizing that their effectiveness is determined by strategic decisions and contextual factors, including historical legacies, current political environments and specific issue characteristics, Shiffman argues persuasively how these networks are likely to achieve better results when they construct compelling framings and build broad strategic coalitions.^2^ Based on this analysis, Shiffman suggests more generally that networks face four challenges in generating attention and resources: problem definition; positioning; coalition-building; and governance.

However, in common with most recent global health governance analysis, Shiffman underplays the important role of states in these global networks in his analysis. Shiffman argues, “The spread of these [global health] networks represents a transformation in the way global health is governed: from a system largely dominated by hierarchical forms of organization—particularly nation-states and inter-state organizations—to one also characterized by horizontal networking and growing participation of non-state actors.” Our commentary questions the basis of this assertion, and poses the question if, instead states’ roles might simply be evolving. As the body which negotiates and signs international agreements, (often) provides the resourcing, and is responsible for implementing and prioritizing initiatives, state recognition and support is a prerequisite to enabling and determining global health networks’ success. The role of states deserves greater attention, analysis and consideration, particularly when considering new or emerging actors like networks. In this response, we first contextualize the underappreciated role of the state within the current conceptualization and discourse on global health. Second, we use the tobacco case study to showcase the decisive role of states in determining progress for global health networks. Third, we highlight how states use a legitimacy loop to gain legitimacy from and provide legitimacy to global health networks.

## The Underappreciated Role of States Within Global Health

Discussions of globalization and global governance continue to grapple primarily with the evolving role of non-state actors in a rapidly changing world.^3^ Definitions remain contested, but global governance generally focuses on the management of challenges previously considered within the domain of a sovereign state, and are now considered unmanageable by single or multiple states.^4^ Global governance and global health share a focus on transnational issues and a need to go beyond the state to address new challenges. Globalization, and the accompanying proliferation of new actors, changed and challenged the role of states within global health governance. During the 2002 SARS situation, for example, the World Health Organization (WHO) assumed and asserted authority over individual states, supporting the perception of major a change in what Fidler called “Westphalian public health.”^5^ This decline of the state and rise of an assertive WHO secretariat supported by global civil society and transnational media networks resonated with scholars seeking to understand a growing shift away from the state. Indeed, some argued that the state was becoming “hollowed out” by globalization^6^ and that the global health governance landscape was so fragmented that states no longer held power over policy-making.^7^ However, reports of the ‘death’ of states within global health governance may be ‘greatly exaggerated.’^8^ While recognizing the rise of new actors and partnerships, the state remains a dominant and decisive actor in global health.

For example, in the SARS case, states did not contest WHO’s assumption of broader powers as SARS containment served their interests; if it had not and threatened their interests, they could have blocked or ignored WHO. For example, just a few years later in 2007, Indonesia did just this. Indonesia refused to share avian influenza samples with the global community.^9^ More recently in the wake of Ebola, 58 states party to the apparently legally binding International Health Regulations (IHR) disregarded their commitments imposing travel restrictions.^10^

There is no doubt that globalization challenges states to evolve. But rather than simply decline, states continue to adapt and respond. States no longer solely reflect national preferences, but instead accommodate both national and international policy demands.^11^ Recent political shifts in the United States and Europe reveal one response to the disenfranchisement felt at national levels from globalization (and the need to accommodate international policy demands) and reflect an attempt to reassert sovereign power. Different ideologies and approaches dictate various state responses to the new reality, but what is clear is that the state is aiming to retain a dominant position, even as it continues to respond to increasing influence and engagement from business, civil society and international institutions.

## The Tobacco Global Health Network

Tobacco, as presented in the global health network case study, exemplifies the challenges globalization posed to states trying to protect their citizens’ health.^12^ The international tobacco industry capitalized on changes in technology and trade liberalization to target emerging markets and expand their business in states with less effective tobacco control.^13^ States responded with the Framework Convention on Tobacco Control (FCTC).^12^ The process to start the FCTC only began once state representatives attending the World Health Assembly approved the process to begin with a resolution.^14^ States like Canada were strong supporters.^15^ Other states such as Brazil^16^ and Thailand,^17^ which had made domestic progress combatting tobacco, viewed the FCTC process as an opportunity to exert soft-power leadership and expand their influence both deepening the consolidation of their own domestic progress against tobacco, and also inspiring other states.

States not only empowered WHO to move forward to negotiate a FCTC, but states also funded and directed WHO in the 1990s to provide resources to facilitate the creation of a civil society alliance to co-ordinate non-governmental organization (NGO) participation in negotiations to ensure the FCTC agreement was approved. States used their ability to direct and fund WHO to support NGOs and research networks creating a global health network to achieve their interests in achieving a treaty. In May 2003, WHO’s 192 member states approved this treaty which entered international law in February 2005. This treaty challenged and shifted state sovereignty, but these changes were state-initiated, state-sponsored, state-approved and state-ratified. Researchers, advocates and policy-makers acting within this network were crucial, but they were also supported, enabled and ultimately sanctioned by states. The FCTC should also serve as reminder of the continued primacy of the state as an actor within global health. More recently, global tobacco companies have sought to challenge states’ ability to enact plain package labelling using international trade agreements; however, states have prevailed, against much of the global health communities’ concern and predictions.

## The Legitimacy Loop Between States and Global Health Networks

The importance of state support for global health networks is also related to legitimacy, where states and global health networks each use the other to legitimize and amplify efforts; establishing a ‘legitimacy loop.’ For example, during the FCTC process, states supported and sanctioned networks as they served their interests, legitimizing their efforts and advancing their positions, as NGOs could take approaches states could not. In other words, states used global health networks as a tool to shift other states’ positions, and legitimize the continued dominance of the state-centric system.

More recently, the conceptualization of the post-2015 development agenda showcases this policy loop. Starting in 2011 and 2012, states determined and established a United Nations’ process whereby states determined the final framework. To legitimize the effort, this process accommodated and included countless consultations with many non-state actors, but this was at the discretion of states and the shape of the consultations controlled by states. States still negotiated and determined the outcome framework. Yet during both the FCTC and post-2015 negotiations, global health networks sought to foster relationships with states to leverage states to legitimize their positions and advance their interests; this was both welcomed and facilitated by states.

## Conclusion

Shiffman provides a valuable service in highlighting the importance of global health networks and how they can be improved. Adding to this foundation, it is critical to recognize the decisive role states play. Moving forward in assessing global health networks’ effectiveness, identifying and further investigating state support as a determinant of success will be critical. Some scholars have argued that global health still needs to be further “globalized.”^18^ Current trends, however, like the recent change to the WHO’s Director-General election process giving all states an equal vote, seem to reflect the opposite: a re-assertion of state power within global health. The question, in the shifting international environment, is how will this continue to evolve? Will states continue to sanction global health networks to advance their interests and fill governance gaps? Will states continue adapting and facilitating innovation within global health capitalizing on new ways to generate ideas, pool resources and enable more shared decision-making processes?^19^ Or will states seek to re-assert their role more forcefully, re-consolidating their power and reversing some changes over the last twenty years?

Of course, states are not unitary actors. State actions and decisions are heavily contested and determined by a number of other national and international non-state actors, global institutions, as well as other states. Analyzing what drives states to commit, prioritize, invest and implement agreements is the critical issue. Understanding how global health networks and states interact and evolve to shape and support their respective interests should be a focus for future research.

## Acknowledgements

The authors thank Johanna Hanefeld for helpful feedback on a draft of this comment.

## Ethical issues

Not applicable.

## Competing interests

Authors declare that they have no competing interests.

## Authors’ contributions

RM conceived and drafted the article. RDS provided critical input and comments.
